# Surgery for degenerative cervical myelopathy in the elderly: a nationwide registry-based observational study with patient-reported outcomes

**DOI:** 10.1007/s00701-022-05282-y

**Published:** 2022-07-19

**Authors:** Tonje Okkenhaug Johansen, Vetle Vangen-Lønne, Siril T. Holmberg, Øyvind O. Salvesen, Tore K. Solberg, Agnete M. Gulati, Øystein P. Nygaard, Sasha Gulati

**Affiliations:** 1 Department of Neuromedicine and Movement Science, Norwegian University of Science and Technology, NO-7491 Trondheim, Norway; 2 Department of Neurosurgery, St. Olavs Hospital, NO-7006 Trondheim, Norway; 3 Department of Public Health and General Practice, Norwegian University of Science and Technology, NO-7491 Trondheim, Norway; 4 Department of Neurosurgery and the Norwegian Registry for Spine Surgery, University Hospital of North Norway, Tromsø, Norway; 5 Institute for Clinical Medicine, UNN The Arctic University of Norway, Tromsø, Norway; 6 Department of Rheumatology, St. Olavs Hospital, NO-7006 Trondheim, Norway; 7 National Advisory Unit On Spinal Surgery, St. Olavs Hospital, NO-7006 Trondheim, Norway

**Keywords:** Myelopathy in the elderly, Degenerative cervical myelopathy (DCM), Cervical spondylotic myelopathy (CSM), Surgery, Neck Disability Index (NDI), European Myelopathy Score (EMS)

## Abstract

**Background:**

The aim of this study was to investigate whether clinical outcomes in patients aged ≥ 70 undergoing decompressive surgery for degenerative cervical myelopathy (DCM) differ from those of younger patients (50–70 years) at 1 year.

**Methods:**

Data were obtained from the Norwegian Registry for Spine Surgery (NORspine). Among 651 patients included, 177 (27.2%) were ≥ 70 years old. The primary outcome was change in the Neck Disability Index (NDI). Secondary outcomes were changes in the European Myelopathy Score (EMS), quality of life (EuroQoL EQ-5D), numeric rating scales (NRS) for headache, neck pain, and arm pain, and complications.

**Results:**

Significant improvements in all patient-reported outcomes (PROMs) were detected for both age cohorts at 1 year. For the two age cohorts combined, there was a statistically significant improvement in the NDI score (mean 9.2, 95% CI 7.7 to 10.6, *P* < 0.001). There were no differences between age cohorts in mean change of NDI (− 8.9 vs. − 10.1, *P* = 0.48), EQ-5D (0.13 vs. 0.17, *P* = 0.37), or NRS pain scores, but elderly patients experienced a larger improvement in EMS (0.7 vs. 1.3, *P* = 0.02). A total of 74 patients (15.6%) in the younger cohort and 43 patients (24.3%) in the older cohort experienced complications or adverse effects within 3 months of surgery, mainly urinary and respiratory tract infections.

**Conclusion:**

Surgery for DCM was associated with significant improvement across a wide range of PROMs for both younger and elderly patients. Surgery for DCM should not be denied based on age alone.

## Introduction

Degenerative cervical myelopathy (DCM), or cervical spondylotic myelopathy, is the most common cause of spinal cord impairment [19] and is associated with a wide range of symptoms and findings including gait disturbances, imbalance, loss of dexterity, impaired coordination, frequent falls, pain and stiffness in the neck, pain and numbness in limbs, and autonomic alterations that may cause bowel, urinary, and sexual problems [1, 28]. The initial symptoms are often subtle, and considerable delay in diagnosis is common. Clinicians need to be aware of the diagnosis and refer to MRI when DCM is suspected. There is growing evidence that decompressive surgery in selected patients can arrest progression of myelopathy and provide meaningful improvements in functional status, neurological outcomes, pain, and quality of life [10, 13]. However, complete resolution of symptoms is unlikely following surgery and risk associated with surgery is not negligible, as almost one in three patients reports adverse events within 3 months [12]. One suggested predictor for a less fortunate outcome after surgery is age [38]. As the elderly segment of the population continues to grow and MRI is readily available, the incidence of DCM is expected to rise. The prevalence and incidence of DCM is not well studied [3]. Studies on the general health of the Norwegian population show that at 65 years of age, life expectancy for men is 18.6 years, of which 14.4 will be healthy life years, and 21.5 years for women, of which 16.7 will be healthy life years. We do, however, not have good numbers for general health and quality of life for persons > 75 years of age [9].

High-quality data on surgical outcomes among elderly patients operated for degenerative cervical myelopathy are scarce [36], and there is concern that elderly patients experience less favorable outcome and more complications compared with younger patients [38]. However, the significance of age on the outcome after DCM surgery remains unclear. The purpose of this study was to compare the effectiveness and safety of surgery for DCM in patients aged ≥ 70 years vs. patients aged 50–70 years. We hypothesized that the elderly cohort would improve less than the younger cohort, and that they would have more complications.

## Methods

The reporting in this paper is consistent with the STROBE (Strengthening the Reporting of Observational Studies in Epidemiology) guidelines [35]. The study was approved by the Regional Committee for Medical Research and Health Research Ethics in Central Norway (2016/840), and all participants provided written informed consent.

### Study population

The Norwegian Registry for Spine Surgery (NORspine) provides prospectively collected data on patients undergoing surgery for degenerative spinal disorders. NORspine is used for quality control and research, and provides data on demographics, lifestyle, and comorbidity, as well as patient-reported outcomes after spinal surgery [27]. All clinics that perform surgery for degenerative cervical disorders in Norway report to the registry [24, 26]. More than 80% of all surgeries on degenerative cervical spine in Norway are registered [13]. The study was planned after the data were collected, but before retrieval of data from the registry. Patients were included in the study if they had a primary diagnosis of DCM, were ≥ 50 years, and had undergone decompressive surgery between January 2012 and June 2018. Patients undergoing surgery for myelopathy for all other reasons, such as trauma, malignancies, infection, or deformity, are excluded from the registry. The patients were dichotomized into patients 50–70 years and patients aged ≥ 70 years.

### Surgical procedures

The patients were referred to surgery based on clinical symptoms of myelopathy and corresponding radiographic findings. The surgical approach, number of operated levels, and the use and type of instrumentation were performed at the surgeons’ discretion.

### Outcome measures

The primary outcome measure was change in Neck Disability Index (NDI) from baseline to 1-year follow-up. Secondary outcome measures were changes in the European Myelopathy Score (EMS), EQ-5D (EuroQoL’s instrument for measuring quality of life), and numeric rating scales (NRS) for headache, neck pain, and arm pain. The Global Perceived Effect (GPE) scale was used to measure the patients’ assessment of their condition 1 year after surgery. In addition, we reported surgeon and patient-reported complications that occurred within 3 months of surgery.

The NDI is a self-rated questionnaire developed for patients with neck disability. It has been translated into Norwegian and tested for psychometric properties [18]. The questionnaire is composed of ten items: seven related to activities of daily living (personal care, lifting, reading, work/daily activities, driving, sleep, and recreation), two related to pain (pain, headache), and one related to concentration. Each item is rated from 0 to 5. The NDI summary score ranges from 0 to 100, with lower scores indicating less disability.

The severity of cervical myelopathy was assessed by using the EMS [14, 34]. The EMS has five subscores: gait (1–5 points), bladder and bowel function (1–3 points), hand function (1–4 points), proprioception and coordination (1–3 points), and dysesthesia and paresthesia (1–3 points) [14, 34]. All subscores are functional, self-rated criteria that do not require formal testing. The total score ranges between 5 and 18. The EMS scores were dichotomized into mild DCM (scores of ≥ 13) and moderate-to-severe (scores between 5 and 12) [13].

Changes in health-related quality of life were measured with EQ-5D. The Norwegian version has shown good psychometric properties [30]. It evaluates five dimensions of the quality of life: mobility, self-care, activities of daily living, pain, and anxiety and/or depression. For each dimension, the patient describes three possible levels of problems (none, mild-to-moderate, and severe). An index value for health status is generated for each patient. Scores range from − 0.6 to 1, where 1 indicates perfect health.

Headache, neck and arm pain were measured with NRS [15]. NRS is a one-dimensional pain scale ranging from 0 (no pain) to 10 (worst imaginable pain).

The GPE scale [20] has seven categories: (1) complete recovery, (2) much better, (3) slightly better, (4) unchanged, (5) slightly worse, (6) much worse, and (7) worse than ever.

Surgeons provided data related to the following perioperative complications: unintentional durotomy, nerve root injury, wrong level of surgery, misplacement of implant, intraoperative hemorrhage requiring blood replacement, respiratory complications, anaphylactic reaction, spinal cord injury, esophageal injury, major vessel injury, cardiovascular complications, and other nerve injury. Patient-reported complications that occurred within 3 months of surgery were superficial wound infection, deep wound infection, urinary tract infection, pneumonia, pulmonary embolism, deep vein thrombosis, dysphagia, and dysphonia.

### Data collection

Patients completed a self-administered questionnaire with baseline data on admission for surgery. The questionnaire included questions about demographics and personal characteristics (marital status, education, body mass index, and smoking). In addition, baseline data on patient-reported outcome measures (PROMs) were collected. Using a standard registration form, surgeons recorded data on diagnosis, comorbidity (including rheumatic diseases, hip or knee osteoarthritis, depression or anxiety, musculoskeletal pain, neurological disorder, cerebrovascular disease, cardiovascular disease, vascular claudication, lung disease, cancer, osteoporosis, hypertension, endocrine disorders), American Society of Anesthesiologists (ASA) score, image-related findings, hospital stay, and surgical procedure. NORspine distributed self-administered questionnaires to the patients by mail 3 and 12 months after surgery, without involving the treating hospitals. Non-responders received one reminder, together with a second copy of the questionnaire.

### Statistical analysis

Statistical analyses were performed with SPSS version 26 (IBM) and Software R version 3.6.3. For statistical comparison tests, the significance level was defined as *P* ≤ 0.05. Frequencies were used for demographic variables at baseline, and changes in EMS, NDI, EQ-5D, and NRS were analyzed with paired sample *T* tests. Independent sample *T* tests were used to compare the changes between the two groups.

Missing data were handled with linear mixed model analyses. This strategy was in line with studies showing that imputations are not necessary before performing linear mixed model analysis of longitudinal data [23, 33]. In the mixed model, patients were not excluded from the analysis if a variable was missing at some, but not all, time points after baseline.

## Results

In total, 651 patients were included in the study. There were 474 (72.8%) patients in the age group 50–70 years and 177 (27.2%) patients aged ≥ 70 years. A total of 525 participants (81%) provided PROMs at 3 and/or 12 months, and the response rate was similar for both age cohorts (80% vs. 84%, *P* = 0.24). Baseline characteristics are presented in Table 1.

**Table 1:** Demographic characteristics, coexisting illness, and measures of health status for both groups

Variable	Age 50–70	Age ≥ 70	*P* value
No. (%)	474 (72.8%)	177 (27.2%)	
Age–year (median, range)	59 (50–69)	74 (70–87)	
Female sex—no. (%)	172 (36.3%)	67 (37.9%)	0.71
Married or partner—no. (%)	334 (70.5%)	112 (64.3%)	0.11
College education—no. (%)	142 (32.0%)	44 (27.3%)	0.27
Mean body mass index	27.5 (95% CI 27.1 to 27.9)	26.0 (95% CI 25.3 to 26.6)	< 0.001
Current smoker—no. (%)	154 (33.1%)	28 (15.8%)	< 0.001
Comorbidity—no. (%)	273 (56.6%)	140 (79.1%)	< 0.001
Cardiovascular disease	51 (10.8%)	64 (36.2%)	
Cerebrovascular disease	12 (2.5%)	10 (5.6%)	
Diabetes mellitus	38 (8.0%)	21 (11.9%)	
Chronic lung disease	42 (8.9%)	20 (11.3%)	
Hypertension	99 (20.9%)	75b(42.4%)	
Osteoporosis	3 (0.6%)	6 (3.4%)	
Chronic neurologic disease	14 (3.0%)	13 (7.3%)	
Chronic musculoskeletal pain	29 (6.1%)	9 (5.1%)	
Cancer	7 (1.5%)	14 (7.9%)	
Rheumatoid arthritis	18 (3.8%)	7 (4.0%)	
Ankylosing spondylitis	7 (1.5%)	0 (0.0%)	
Other rheumatic disease	12 (2.5%)	8 (4.5%)	
Prior cervical spine surgery	65 (13.7%)	18 (10.2%)	0.23
Symptoms > 1 year	98 (21.5%)	31 (18.7%)	0.43
ASA grade > 2	78 (17.0%)	101 (59.1%)	< 0.001
Preoperative NDI	33.1 (95% CI 31.5 to 34.7)	35.6 (95% CI 32.5 to 38.7)	0.08
Preoperative EMS	14.5 (95% CI 14.3 to 14.7)	12.8 (95% CI 12.3 to 13.3)	< 0.001
EMS moderate-to-severe (5–12 points)	77/418 (18.2%)	64/154 (41.6%)	< 0.001
Preoperative EQ-5D	0.47 (95% CI 0.44 to 0.50)	0.40 (95% CI 0.34 to 0.46)	0.01

Surgical outcomes are listed in Table 2. For both cohorts combined, there was a statistically significant improvement in NDI score (mean change 9.2, 95% CI, 7.7–10.6, *P* < 0.001). Complete case analyses showed no difference between age cohorts in change in NDI (mean difference 1.3, 95% CI − 2.2–4.7, *P* = 0.48), EQ-5D (mean difference − 0.04, 95% CI − 0.11–0.04, *P* = 0.37), NRS headache (mean difference 0.2, 95% CI − 0.6–0.9, *P* = 0.64), NRS neck pain (mean difference 0.5, 95% CI − 0.2–1.2, *P* = 0.18), or NRS arm pain (mean difference 0.5, 95% CI − 0.5–1.1, *P* = 0.44) from baseline to 1 year. EMS measured on the whole cohort changed from 14.0 to 14.9 (mean change 0.9 (95% CI 0.7–1.1, *P* < 0.001). Elderly patients had lower EMS scores at both baseline (14.5 vs. 12.7, *P* < 0.001) and 1 year (15.2 vs. 14.0, *P* < 0.001), and moderate-to-severe DCM was more common in the elderly group (41.6% vs. 18.2%, *P* < 0.001) compared to younger patients. The mean EMS change was slightly larger in the older age cohort (mean difference 0.6, 95% CI 0.1–1.1, *P* = 0.02).

**Table 2:** Outcomes at 1 year in patients operated for degenerative cervical myelopathy

Complete case analyses (*N* = 363)								
	Age 50–70 years, *N* = 267			Age ≥ 70 years, *N* = 96				
Variable	Baseline	1 year	Mean change	Baseline	1 year	Mean change	Difference in mean change between groups (95% CI)	*P* value
NDI	33.2	24.3	− 8.9	36.1	26.0	− 10.1	1.3 (− 2.2 to 4.7)	0.48
EQ-5D	0.48	0.62	0.13	0.41	0.58	0.17	− 0.04 (− 0.11 to 0.04)	0.37
EMS	14.5	15.2	0.7	12.7	14.0	1.3	− 0.6 (− 1.1 to − 0.1)	0.02
Neck pain NRS	4.5	2.9	− 1.6	4.6	2.5	− 2.1	0.5 (− 0.2 to 1.2)	0.18
Arm pain NRS	5.1	3.5	− 1.5	5.0	3.2	− 1.8	0.5 (− 0.5 to 1.1)	0.44
Headache NRS	3.1	2.1	− 1.0	3.2	2.0	− 1.1	0.2 (− 0.6 to 0.9)	0.64
Mixed linear model analyses (*N* = 651)								
	Age 50 – 70 years, *N* = 474			Age ≥ 70 years, *N* = 177				
Variable	Baseline	1 year	Mean change	Baseline	1 year	Mean change	Difference in mean change between groups (95% CI)	*P* value
NDI	33.3	24.3	− 8.2	36.3	26.7	− 9.6	1.4 (− 2.0 to 4.8)	0.42
EQ-5D	0.47	0.61	0.14	0.39	0.57	0.17	0.03 (− 0.1 to 0.03)	0.32
EMS	14.4	15.1	0.7	12.7	13.9	1.3	− 0.6 (− 1.0 to − 0.2)	0.01
Neck pain NRS	4.5	2.9	− 1.6	4.4	2.4	− 2.1	0.5 (− 0.1 to 1.1)	0.11
Arm pain NRS	5.0	3.5	− 1.5	4.8	3.1	− 1.7	0.2 (− 0.4 to 0.8)	0.56
Headache NRS	2.9	2.0	− 0.9	2.8	1.8	− 0.9	0.0 (− 0.6 to 0.6)	0.97

The results of linear mixed model analyses were similar to those of the complete case analyses for all PROMs.

**Fig. 1:**
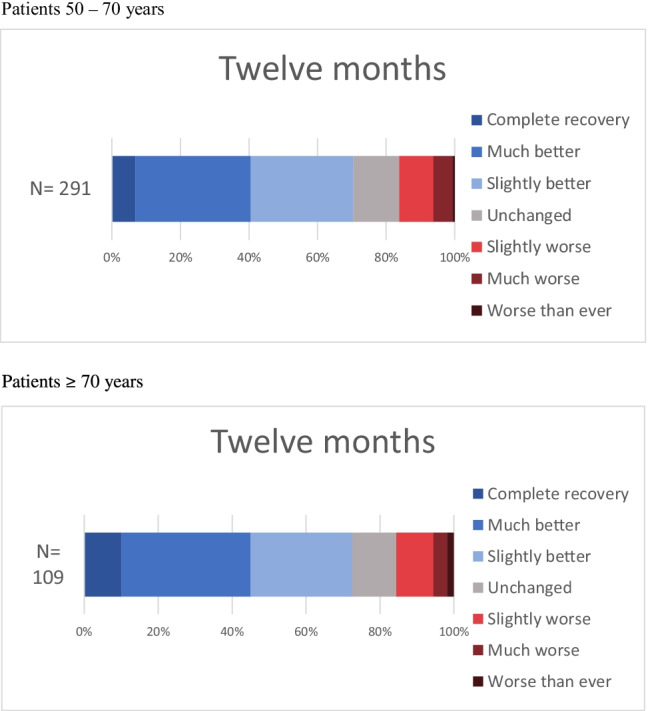
Patients’ global perceived effect of surgery

GPE for both age groups combined revealed that 167 out of 400 patients (41.8%) reported either complete recovery or much better at 1 year, and 64 out of 400 (16%) reported that they were slightly worse, much worse, or worse than ever at 1 year (Fig. 1). There was no difference in perceived benefit at 3 (*P* = 0.34) or 12 (*P* = 0.39) months.

Most patients were operated via either anterior or posterior approach, and very few underwent circumferential surgery and/or instrumentation. In the older age cohort, 132 patients (74.6%) were operated via posterior approach versus 207 (43.7%) in the younger age cohort.

Patients aged ≥ 70 years had 0.5 days longer hospital stay, mean 2.3 days (CI 95% 2.0 to 2.5) vs. 1.8 days (CI 95% 1.6 to 1.9) in the younger age cohort, *P* = 0.001.

Table 3 provides details of surgeon and patient-reported complications. There were no deaths within 30 days of surgery and no differences in surgeon-reported complications. In total, 117 patients (18.0%) reported complications within 3 months of surgery, 74 (15.6%) in the younger cohort vs. 43 (24.3%) in the older age cohort. Patients aged ≥ 70 years reported more urinary and respiratory tract infections within 3 months of surgery compared with patients in the younger age group.

**Table 3:** Surgical treatments, complications, and events

Variable	Age 50–70 years	Age ≥ 70 years	Mean difference (95% CI)	*P* value
Surgical approach				
Anterior	265 (55.9%)	45 (25.4%)	0.04 (0.22 to 0.39)	< 0.001
Posterior	207 (43.7%)	132 (74.6%)	− 0.31 (− 0.39 to 0.23)	< 0.001
Instrumented fusion	10 (2.1%)	6 (3.4%)	− 0.01 (− 0.04 to 0.01)	0.35
Circumferential	2 (0.4%)	0 (0.0%)	0.00 (− 0.01 to 0.01)	0.39
Number of levels decompressed, median (range)	2 (1–5)	2 (1–6)		
Patients with complications, no. (%)	74 (15.6%)	43 (24.3%)	− 0.09 (− 0.15 to − 0.02)	0.01
Perioperative complications, no. (%)	5 (1.1%)	5 (2.8%)	− 0.02 (− 0.04 to 0.00)	0.10
Unintentiontal durotomy	1 (0.2%)	2 (1.1%)		
Nerve root injury	0 (0.0%)	0 (0.0%)		
Iatrogenic spinal cord injury	1 (0.2%)	0 (0.0%)		
Wrong level surgery	0 (0.0%)	0 (0.0%)		
Misplacement of implant	0 (0.0%)	0 (0.0%)		
Esophageal injury	0 (0.0%)	0 (0.0%)		
Major blood vessel injury	0 (0.0%)	0 (0.0%)		
Postoperative hematoma	0 (0.0%)	0 (0.0%)		
Cardiovascular complications	0 (0.0%)	1 (0.6%)		
Respiratory complications	0 (0.0%)	1 (0.6%)		
Anaphylactic reaction	3 (0.6%)	0 (0.0%)		
Other complications	3 (0.6%)	1 (0.6%)		
Patient-reported complications within 3 months, no. (%)	72 (19.6%)	40 (27.4%)	− 0.08 (− 0.16 to 0.01)	0.05
Deep wound infection	6 (1.6%)	3 (2.1%)	− 0.04 (− 0.03 to 0.2)	0.74
Superficial wound infection	20 (5.4%)	9 (6.2%)	− 0.01 (− 0.1 to 0.04)	0.75
Urinary tract infections	16 (4.3%)	22 (15.1%)	− 0.10 (− 1.6 to − 0.1)	< 0.001
Pneumonia	5 (1.4%)	7 (4.8%)	− 0.03 (− 0.1 to − 0.001)	0.02
Pulmonary embolism	3 (0.8%)	2 (1.4%)	− 0.06 (− 0.24 to 0.13)	0.56
Deep venous thrombosis	5 (1.4%)	2 (1.4%)	0.00 (− 0.02 to 0.02)	0.99
Dysphagia total	32 (8.7%)	14 (9.6%)	− 0.01 (− 0.10 to 0.10)	0.94
Anterior approach	26 (13.0%)	9 (22.5%)	− 0.10 (− 0.22 to 0.03)	0.12
Posterior approach	5 (3.1%)	5 (4.9%)	− 0.02 (− 0.06 to 0.03)	0.47
Dysphonia	32 (8.7%)	16 (11.0%)	− 0.02 (− 0.1 to 0.03)	0.43

Dysphagia was reported by 32/368 (8.7%) in the younger age cohort and 14/146 (9.6%) in the older age cohort.

## Discussion

Patients aged ≥ 70 years experienced similar change in PROMs after decompressive surgery compared to younger patients. Moreover, elderly patients reported a larger improvement in EMS compared to younger patients. Elderly patients had longer hospital stays and experienced more minor complications compared with those aged 50–70 years.

Patients should be informed that complete resolution of symptoms and disability is unlikely, and that there is a risk of complications and neurological deterioration following surgery for DCM. Elderly patients had higher disability at baseline, and this may explain more residual symptoms at 1 year. Still, almost three out of four patients in both age cohorts reported at least some improvement according to GPE assessments at 1 year. Both groups experienced statistically significant improvement in all outcome measures. The clinical improvement is, however, modest, and the study population as a whole did not meet the recently suggested MCIC for NDI, NRS pain scores, and EQ-5D in a study from the Swedish Spine Registry [29]. One could argue that MCIC is of lesser importance given the often progressive nature of DCM [7].

The choice of cutoff age for “elderly” varies between studies. Some define elderly as > 65 years of age [25] and others as > 75 years of age [21]. A study trying to define a cutoff age for what is considered elderly by interviewing 300 individuals landed on 73.7 years [6]. In a recent AOSpine study on DCM surgery in the elderly, the cutoff age for elderly was set to 70 years [37]. In concordance with this, we chose 70 years of age or older as our definition of elderly.

Cervical spinal degeneration and comorbidity increase with age, and as expected, the two cohorts were not balanced for baseline factors. Patients in the older age cohort had more disability at baseline and at 1 year after surgery compared with patients in the younger age cohort. Still, the older age cohort had similar improvement in all PROMs at 1 year despite more comorbidity and higher ASA grade which possibly could impact the results in a negative manner. The proportion of patients operated for moderate-to-severe myelopathy was significantly higher in the older age cohort. Patients in both age groups who were operated for moderate-to-severe myelopathy experienced a larger improvement measured in EMS than those operated for mild myelopathy.

When comparing the study population to the general population, we find that the younger study population has somewhat higher prevalence of DMII and chronic obstructive lung disease and somewhat lower prevalence of stroke, hypertension, and cancer than the general population. The older age cohort has somewhat higher prevalence of chronic obstructive lung disease and somewhat lower prevalence of hypertension and cancer than the general population [16]. The numbers and different disease categories are not directly comparable, so statistical analysis is not sensible, but the numbers indicate that the populations studied are not very different from the general population.

In recent years, studies on frailty have been published, and one can argue that frail vs. non-frail patients are a better comparison than young vs. old patients. Frailty is defined as reduced physiological reserve which means that patients are susceptible to sudden, disproportionate functional decline following stressor events [8]. Age is incorporated in many of the frailty assessment scales, and frailty is strongly associated with age, but should be viewed as a separate entity [4]. Only patients that have been operated are included in NORspine. All these patients have been through preoperative screening, and those deemed unfit for surgery are already excluded. This would comprise many of the frail patients with cardiovascular challenges believed to be a risk factor for surgery. One must bear in mind that frailty is a dynamic entity which can both worsen and improve [17], that DCM patients score higher on frailty scales due to the nature of their diagnosis, and that improvement in DCM symptoms means improvement in frailty. Data from NORspine do not comprise frailty scales, but baseline data including comorbidity and ASA grade do not imply less frailty in the elderly group, so this does not weaken our results or conclusion.

Results from studies on elderly patients undergoing surgery for DCM vary (Table 4). A previous study has shown that age is a predictor of less favorable outcomes after surgery for DCM [38]. This contrasts to our study that demonstrated improvement, also in patients in the age group 70 years or older. They discuss whether this is due to age-related changes in the spinal cord, general degeneration associated with normal aging, and comorbidity. Results from the AOSpine CSM study show that elderly patients experience improvement, but not to the same extent as patients aged < 70, and that they have a similar complication rate as younger patients [37]. Similar improvement in PROMs were also observed in a recent study comparing different age groups [5].

**Table 4:** Overview over studies on surgical outcome after surgery for DCM in the elderly

Author/year	Study design	Aim	Results	Complications
Zhang et al., *Spinal Cord*, 2016	Prospective cohort study	Characterize risk factors for poor surgical outcome in DCM patients	Advanced age, long duration of symptoms, and intramedullary changes on MRI were risk factors for poor outcome	Not reported
Nakashima et al., *J Neurol Neurosug Psychiatry*, 2016	Prospectively enrolled patients operated for DCM in the CSM-International study	Determine whether age is an independent predictor of surgical outcome for DCM	Both groups had significant improvement across all outcome measures. Elderly patients (≥ 65 years of age) had significantly lower mJOA and Nurick scores at both baseline and 2 years follow-up	No difference between the age groups. A greater percentage of elderly experienced screw malposition in the perioperative period
Madhavan et al., *Neurosurg Focus*, 2016	Meta-analysis	Compare outcome after surgery for degenerative cervical myelopathy for patients ≥ 75 years of age compared with younger patients	Elderly patients (≥ 75 years of age) had lower mJOA scores both before and after surgery	Elderly patients experienced delirium more often than younger patients
Wilson et al., *J Clin Med*, 2019	Ambispective, propensity-matched, multicenter study	Evaluate effect of age on functional and QoL outcomes after surgery for degenerative cervical myelopathy	Both younger (< 70 years of age) and elderly patients (≥ 70 years of age) showed significant improvements in mJOA score, NDI score, and SF36-PCS at 2 years, but the improvement was larger for mJOA and SF-36 PCS in the younger age cohort	No significant differences between age cohorts
Croci et al., *J Neurosurg Spine*, 2022	Multicenter registry-based study	Compare functional outcome and QoL outcome in patients < 65, 65–74, and ≥ 75 years of age	Younger patients (< 65 years of age) had significantly worse NDI and lower EQ-5D VAS and EQ-5D at baseline compared with early and late elderly patients (65–74 and ≥ 75 years of age). On unadjusted analysis at 3 months, younger patients had greater improvement on VAS arm pain, NDI, and EQ-5D VAS compared with early and late elderly patients, but on adjusted analyses at 12 months, there were no differences in patient-reported outcomes	Only return to operating room and 30-day mortality were reported. There was no difference in reoperation rate or 30-day mortality

*DCM* degenerative cervical myelopathy, *mJOA* modified Japanese Orthopaedic Association scale, *EQ-5D* EuroQoL five dimensions, *NDI* neck Disability Index, *SF36* short form 36, *PCS* physical component score, *VAS* visual analog scale

In our study, the surgeons decided upon surgical approach based on patient factors, clinical symptoms and findings, and radiographic imaging. Elderly patients were more likely to undergo posterior decompression (Table 2). It would be interesting to compare the effectiveness of different surgical treatments, but this was beyond the scope of our study. The authors of a recent RCT comparing ventral vs. dorsal surgery for DCM concluded that there were no significant differences in outcomes, but more complications in the ventral surgery group [12] mainly due to dysphagia. We found similar incidence rates for dysphagia in both the younger and older age cohorts. The proportion of dysphagia was a bit higher among elderly patients operated via anterior approach, but this did not reach statistical significance. Interestingly, some patients that were operated via posterior approach also reported dysphagia at 3 months after surgery. This suggests that other factors than manipulation with the esophagus during surgery might play a role in the development of dysphagia.

Total complications were higher in the older age cohort mainly due to urinary and respiratory tract infections within 3 months. This is in line with previous reports on the outcomes of degenerative lumbar spine surgery [11, 22] and the recent AOSpine article on surgery for DCM in the elderly [37]. Life-threatening complications were fortunately rare for both age cohorts.

On average, patients aged ≥ 70 years had 0.5 days longer hospital stays than those in the 50–70 age group. The reasons are probably multifactorial and might include the possibility that fewer patients in the old age group had a life partner or spouse, differences in comorbidity, or different routines for postoperative mobilization, pain management, and hospital discharge. In addition, there were more posterior surgical approaches in the older cohort that probably reflect that degeneration of the spine increases with age and may imply more postoperative pain.

DCM is a frequently encountered diagnosis in spine surgery practice, but the time from symptom debut to surgical assessment is often long. One of the risk factors for poor surgical outcome is long duration of symptoms [38]. In one study, the mean time from symptom debut to diagnosis exceeded 2 years [2]. Approximately 20% of our patients had duration of symptoms more than 1 year. Patients should be identified and promptly referred for MRI and to a spine specialist for surgical assessment. Delayed diagnosis can lead to neurological deterioration and unnecessary residual symptoms because of delayed surgery [32].

### Strengths and limitations

The pragmatic study design based on prospective registry data in an everyday clinical setting with a large study population ensures high external validity. Another strength is the wide range of patient-reported outcome measures. However, the two age cohorts were not balanced for all baseline and treatment factors, as cervical spine degeneration and comorbidity increase with age, and confounding factors were not explored. The main limitation was the loss to follow-up of 19%. However, a previous study from the NORspine registry found no differences in outcomes between responders and non-responders [31]. Another limitation is that patients in the older age cohort are carefully selected for surgery and might not be representative of the total population of elderly DCM patients. NORspine only includes patients that actually undergo surgery, and unfortunately, we do not have any information about patients ineligible for surgical treatment due to frailty and comorbidity. Patient characteristics, indications, and surgical strategies may vary between institutions and countries, and results from our study might differ from other countries and clinical settings.

## Conclusions

Patient-reported outcomes following surgery for DCM showed that patients aged ≥ 70 years experienced similar improvements when compared to younger patients (50–70 years). Hospital stays were slightly longer for those in the elderly age group, but there were no relevant differences in perioperative complications between the two age groups. Patients who were ≥ 70 years of age were more likely to report minor complications within 3 months. Thus, age alone should not be a general contraindication for surgical treatment of DCM.

## Data Availability

No additional data are available.

## Abbreviations

DCM: Degenerative cervical myelopathy
NORspine: Norwegian Registry for Spine Surgery
NDI: Neck Disability Index
EMS: European Myelopathy Score
EQ-5D: EuroQoL five dimensions
NRS: Numeric rating scale
PROM: Patient-reported outcome measure
MRI: Magnetic resonance imaging
STROBE: Strengthening the Reporting of Observational Studies in Epidemiology
GPE: Global Perceived Effect
ASA: American Society of Anesthesiologists
SPSS: Statistical Package for the Social Sciences

## Declarations

### Transparency

The lead author (TOJ) confirms that this paper is an honest, accurate, and transparent account of the reported study, that no important aspects of the study have been omitted, and that any discrepancies from the study as planned have been explained.
